# The physiological and pharmacological effects of *Ziziphoratenuior *L.: A review study

**DOI:** 10.22038/AJP.2021.48607.2606

**Published:** 2022

**Authors:** Ali Zarei, Saeed Changizi-Ashtiyani, Behnam Masmouei, Fatemeh Rasekh, Mansour Sokhandani, Fridoon Jahangir

**Affiliations:** 1 *Department of Physiology, Estahban School of Paramedical Sciences, School of Nursing Hazrat Zahra (P.B.U.H) Abadeh, Shiraz University of Medical Sciences, Shiraz, Iran*; 2 *Department of Physiology, Arak University of Medical Sciences, Arak, Iran*; 3 *Department of Nursing, School of Nursing Hazrat Zahra (P.B.U.H) Abadeh, Shiraz University of Medical Sciences, Shiraz, Iran*; 4 *Department of Biology, Payam Noor University of Tehran, Tehran, Iran *

**Keywords:** Ziziphoratenuior L., Pulegone, Antioxidant, Diabetes

## Abstract

**Objective:**

Many reports have revealed preventive and therapeutic effects of *Ziziphoratenuior*; however, few systematic reviews have evaluated such effects. The present study reviews the physiological and pharmacological effects of *Z. tenuior* extract and its components.

**Materials and Methods:**

English articles were searched in international databases, such as Embase, Scopus, and PubMed; Persian studies were also searched in national databases such as SID and Magiran.

**Results:**

Pulegone is one of the most important effective compounds of *Z. tenuior*, which has analgesic, anti-inflammatory, and anti-stress properties as it affects serotonergic and opioidergic systems and decreases the gastric acid secretion. Moreover, this compound inhibits cholesterol absorption and synthesis, resulting in hyperlipidemic effects and weight loss. In addition to its antioxidant, antitumor, and antibacterial properties, this herb contains an antidiabetic effect mediated by increasing the number of pancreatic beta cells and insulin secretion, and inhibiting alpha-amylase. Although its effective dosage has no side effects, the overuse of its effective compounds, such as pulegone, may raise some liver and pulmonary disorders.

**Conclusion:**

*Z. tenuior *and its extract can have preventive and therapeutic effects on diabetes and hyperlipidemia-associated diseases. Since most studies on this herb were *in vivo*, it is necessary to design clinical trials to evaluate its effects.

## Introduction

Traditional medicine is a complementary therapy system that provides a healthy and preventive lifestyle based on individuals’ characteristics and has therapeutic potentials in various organ disorders. The advantages of using medicinal plants are superior to its disadvantages such as drug resistance, side effects and complex drug interactions, and their high price (Changizi-Ashtiyani et al., 2017 ▶; Zarei et al., 2015a ▶). Moreover, numerous studies have been carried out worldwide to extract bioactive compounds from widely-used medicinal herbs and produce drugs to prevent and treat some diseases (Kolangi et al., 2017 ▶). 

**Figure 1:**
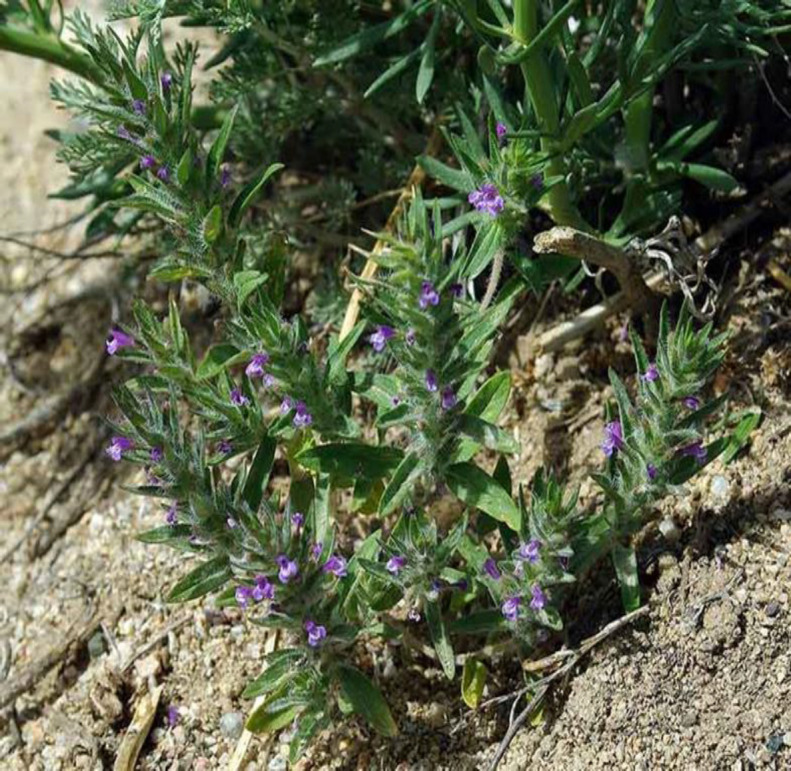
*Ziziphoratenuior* L.

*Ziziphoratenuior* (“*Kakuti*” in Persian) belongs to the Lamiaceae family and has small leaves and white, pink, and purple flowers (Figure 1). Main compounds of *Ziziphora *include pulegone, 3', 5'-dihydroxy acetophenone, isomentone, 2-methyl-5-(1-methyl-ethyl)-phenol, limonene, 12 acetyl-4, 4 d-methyl-cyclopentane-2-isen (Pakniyat and Mousavi, 2014 ▶), ziziphorins (Mehmood et al., 2010 ▶), anthocyanins, proteins, and oils (Zarei et al., 2014 ▶). Pulegone is one of the bioactive compounds of this herb, which is used in the treatment of fever, dysmenorrhea, and gastric tonus owing to its analgesic and anti-inflammatory effects. The steroid compounds, carvacrol, phytonutrients limonene, and alfa-terpineol in the herbs of this family also have analgesic properties (Zendehdel and Babapour, 2010 ▶). This herb encompasses hypolipidemic properties and improves oxidative stress in rabbits lung and liver (Karimi et al., 2013 ▶). Studies on other species of this genus have revealed that these herbs have antidiabetic properties, improve cognitive processes by having positive effects on neural function, such as Alzheimer's disease (Mohammadi and Mohammadi Mehdiabadi Hassani, 2017 ▶; Mohammad Sadeghi et al., 2015 ▶). Moreover, *Ziziphoratenuior *has antitumor (Mohammadi and Mohammadi Mehdiabadi Hassani, 2017 ▶) and antibacterial properties and is used in dairy foods as a flavoring and preservative constituent (Abdolshahi et al., 2018 ▶). As these effects have been reported in a large number of studies, the present study aimed to review the physiological and pharmacological effects of bioactive compounds obtained from *Z. tenuior*. 

## Materials and Methods

**Figure 2:**
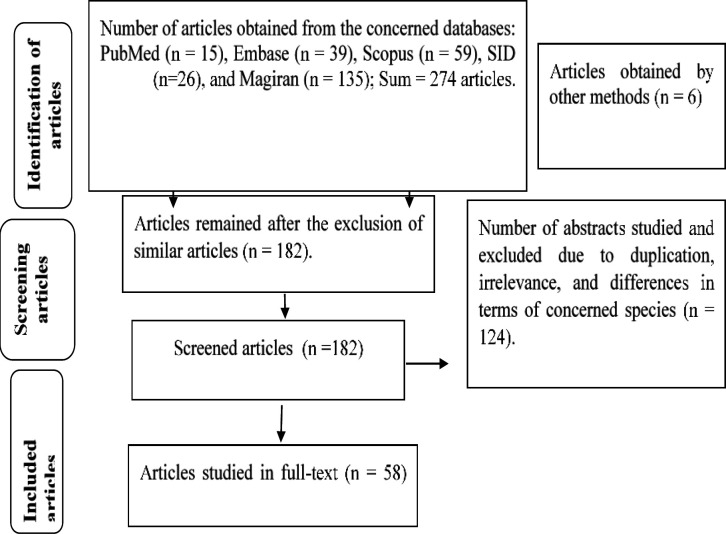
The process of searching and extracting articles on the therapeutic compounds of *Ziziphoratenuior*L

This review study was conducted by searching some reliable international databases, such as Embase (39 records), Scopus (59 records), and PubMed (15 records), as well as Iranian databases, *viz*. SID (26 records) and Magiran (135 records), using some relevant keywords as *Ziziphoratenuior, *Pulegone, Disease, herbal medicine, *Kakuti*on January 15, 2020. Six studies were extracted using other methods such as searching references of the articles. In total, 280 articles were collected for this review. Duplicate studies (98 records), unrelated articles, and those addressing other genera (123 records) were excluded, and totally 58 articles were included in this review (Figure 2). 

## Results

**Antidepressant effect**

Depression is considered one of the main causes of disability worldwide and plays a critical role in the global burden of disease at all ages (Arthur et al., 2020 ▶). Researchers used Forced Swimming Test (FST) and Tail Suspension Test (TST) to evaluate the antidepressant effects of hydroalcoholic extracts obtained from *Z. tenuior*, fluoxetine, and imipramine on immobility, climbing, and swimming *in vivo*. The findings indicate that different doses of the extract decreased the duration of immobility in the FST and TST. In other words, exposing animals to the *Z. tenuior* extract in a dose-dependent manner increases the duration of swimming, similar to that of fluoxetine. Since fluoxetine is also a serotonin reuptake inhibitor, this extract probably reinforces the serotonin neurotransmission. Although few studies have evaluated the antidepressant effects of this extract, some existing data confirm such an effect (Gharetapeh et al., 2014 ▶). 

**Analgesic and wound healing effects**

Currently, pain is controlled using non-steroidal anti-inflammatory drugs (NSAIDs) and opioid analgesic drugs, which have some side effects. Accordingly, it is of paramount importance to achieve analgesic drugs with fewer side effects (Zendehdel and Babapour, 2010 ▶). Zendehdel et al. (2010) ▶ evaluated the analgesic effect of the *Z. tenuior* extract in mice in comparison to indomethacin. The findings indicated that this extract along with indomethacin as a co-treatment decreased visceral pain; however, pretreatment with naloxone and cyproheptadine relatively inhibited the analgesic effects of the extract. The analgesic properties of this herb are probably mediated by serotonergic and opioidergic mechanisms, confirming the use of this herb as an analgesic. The decreased analgesic effects of this extract after pretreatment with naloxone and cyproheptadine result from the activity of naloxone as an opioid receptor antagonist (receptor M) and cyproheptadine as a serotonin receptor blocker (5-HTI). The presence of pulegone, steroid compounds, carvacrol, phytonutrients, limonene, and alfa-terpineol in this herb raises analgesic properties (Zendehdel and Babapour, 2010 ▶). The analgesic effects of pulegone and limonene obtained from this family were reported to be more than those of its monoterpenes (de Sousa et al., 2007 ▶). 

Abbasi et al. (2018) evaluated the effect of Z. tenuior ethanolic extract on the morphine withdrawal signs in mice and found that bruxism, jumping, and diarrhea decreased in the animals. Accordingly, this extract decreases some morphine withdrawal behaviors such as rearing, climbing, writing, and wet-dog shakes (Abbasi et al., 2016). According to another study, the inflammation after inducing ear edema via Xylenol was lowered by intraperitoneal injection of the *Z. tenuior *extract, thereby acting similar to dexamethasone (Ghorbani Ranjbary et al., 2016 ▶).

Compared the analgesic effects of the hydroalcoholic extract and diclofenac using the formalin test and noticed that the level of pain decreased in the group received both the extract and diclofenac. Moreover, pretreatment with naloxone decreased the analgesic effect of the extract (Ahmadi Moghadam and Shahraki, 2015 ▶). The effect of *Z. tenuior* extract was evaluated on the gastric ulcer induced by acetic acid in rats. Results showed an increase in the number of ulcer neutrophils and macrophages and a decrease in the number of fibroblasts (Mousavi Mobarake et al., 2013 ▶). Pulegone and 1, 8-cineole, as the main bioactive compounds in *Z. tenuior* extract, decreased the secretion of gastric acid (Azadmehr et al., 2014 ▶). Flavonoids pass through the blood-brain barrier, control the pain, and lead to an analgesic effect by affecting the Gamma aminobutyric acid (*GABA*), opioid, and alpha 2-adrenergic receptors and inhibiting enzymes contributing to brain inflammation (Table 1) (Mohammadi and Mohammadi Mehdiabadi Hassani, 2017 ▶).

**Hypolipidemic effects**

Hyperlipidemia refers to the increased levels of lipid and cholesterol in plasma, leading to various disorders, including coronary artery diseases (Changizi-Ashtiyani et al., 2018 ▶; Zarei et al., 2015c ▶). Karimi et al. (2013) ▶ evaluated the hypolipidemic effects of aromatic water (AW) obtained from *Z. tenuior *in rabbits receiving a high-cholesterol diet and reported that such a diet increased levels of triglycerides (TG), high-density lipoprotein-cholesterol (HDL-C), very low-density lipoprotein-cholesterol (VLDL-C), atherogenic index (AI), and coronary heart disease index (CHD) in the control group. However, the groups receiving a high-cholesterol diet along with the extract of the herb for four weeks revealed decreased levels of these factors (Shahnazi et al., 2016a ▶; Karimi et al., 2013 ▶). Probably, this herb inhibits intestinal cholesterol uptake, down-regulates LDL receptors, increases the excretion of cholesterol and bile acids via the stool, and decreases cholesterol synthesis (Karimi et al., 2013 ▶). *Z. tenuior* extractalso raises hypertriglyceridemia by inhibiting hepatic lipogenesis through decreasing sterol receptor element-binding protein-1. In other words, it induces lipid oxidation in the liver and muscles by activating proliferator-activated receptors (PARs). Consequently, the conversion of glucose to glycogen is associated with the down-regulation of hepatocyte nuclear factor-4-alpha (HNF-4 α) (Shahnazi et al., 2016 ▶).

Pulegone, isomenthone, 8-hydroxy delta4 (5)-pmen3-one, and limonene are the main compounds found in the essential oil* of Z. tenuior, *the most significant of which is pulegone. Pulegone and its metabolites, including menthofuran, are known as hepatotoxic compounds and lead to pulmonary edema, centrilobular necrosis, and internal bleeding (Karimi et al., 2013 ▶). Nevertheless, a high level of pulegone was noticed in the AW of this herb compared to other hypolipidemic compounds such as limonene. Accordingly, further research should investigate the effects of AW on improving lipid profiles (Karimi et al., 2013 ▶). Evaluating the effects of *Z. tenuior *extract on biochemical factors, including liver function tests and fat profiles, revealed that cholesterol, Alanine aminotransferase (*ALT*) , and TG levels decreased in groups receiving this extract (Dehkordi et al., 2014 ▶). *Z. tenuior* essential oil contains thymol and carvacrol, leading to a decrease in the concentrations of plasma TG (Lee et al., 2003 ▶) and cholesterol in animals by inhibiting the enzyme 3-*hydroxy-3-methylglutaryl coenzyme A* (*HMG*-*CoA*) reductase (Elson and Qureshi, 1995 ▶; Case et al., 1995 ▶). Carvacrol also stimulates the reproduction and growth of lactobacilli, thereby playing an important role in improving blood factors and reducing serum fats (Tschirch, 2000 ▶). Lacobacilli can metabolize and uptake cholesterol in the small intestine and reduce its absorption via the blood (Percival, 1997 ▶). This herb not only has no hepatotoxic effects but also improves hepatic function by causing a significant decrease in alanine aminotransferase (ALT) and a relative but not significant decrease in aspartate aminotransferase (AST) (Dehkordi et al., 2014 ▶).

**Effect**s **on the liver and lungs**

Antioxidant compounds promote the health of the liver due to the significance and exposure of this organ to many oxidizing and carcinogenic agents (Taheri et al., 2012 ▶; Rezaei et al., 2013 ▶). Yazdanzadeh et al. (2017) ▶ evaluated the protective effects of *Z. tenuior* extract on the liver and lung toxicity caused by chlorpyrifos (CPF, a widely-used organophosphate pesticide) in rats. The antioxidant activity of the extract improved the oxidative stress in the liver and lungs; therefore, it seems that CPF toxicity originates from oxidative stress, inducing cell death. This extract protects pulmonary and liver tissues from damages caused by CPF (Yazdinezhad et al., 2017 ▶). 

The effect of *Z. tenuior*hydroalcoholic extract was investigated on cadmium toxicity in the liver and increases were noticed in the activities of the liver enzymes, superoxide dismutase (SOD) and glutathione reductase (GR), in the cadmium group compared to the control group. However, there was a decrease in malondialdehyde (MDA) levels in the control group (Jafari Sani, 2019 ▶).

**Effects on diabetes**

Currently, diabetes as a factor leading to the disability of patients and their hospitalization, imposes significant financial pressure on society (Cho et al., 2018 ▶; Taleb et al., 2017 ▶). The existing methods, such as dietary changes and oral hypoglycemic agents, used to treat type 2 diabetes are accompanied by some limitations. The use of herbs for the treatment of diabetes is highly common in Central Asia (Mohammad Sadeghi et al., 2015 ▶; Case et al.,1995 ▶; Zarei et al., 2015b ▶). Mohammadi et al. (2017) ▶ studied the effect of *Z. tenuior* extract on memory disorders in diabetic rats and reported that this extract prevented spatial memory loss in these animals (Mohammadi and Mohammadi Mehdiabadi Hassani, 2017 ▶). 

The genus *Ziziphora* has antibacterial properties and its extract increases the number of beta cells and eventually insulin levels, and decreases the level of blood sugar in diabetic rats (Mohammad sadeghi et al., 2015 ▶). Sadeghi et al. showed that another species in this genus, called *Z.clinopodioides*, reduced the level of serum glucose in diabetic rats. *Z. clinopodioides* lam could reduce serum glucose level in diabetic animals by increasing insulin secretion (Sadeghi et al., 2015 ▶) and decreased glucose reabsorption in the kidney (Table 1) (Ghafari et al., 2006 ▶, Tian et al., 2011 ▶).

**Table 1:** Some physiological effects of *Ziziphoratenuior* extract at different doses

**Ref.**	**Observed effects**	**Experimental model and animal **	**Dosage/Treatment day**	**Extract **
	Increased number of beta cells and insulin secretory activity	*In vivo*, albino bulb/C mice	100, 150, 200, and 300 mg/kg – 18 days	Hydroalcoholic extract
	Reduced response to visceral pain	*In vivo*, albino N-MRI mice	50, 75, and100 mg/kg	Hydroalcoholic extract
Abbasi et al., 2016	Decreased morphine withdrawal symptoms	*In vivo*, albino N-MRI mice	50, 75, and100 mg/kg	Hydroalcoholic extract
	Increased CD40 surface expression, increased IL12 and IL10 production	*In vitro*	100 μg/ml,	Ethanolic extract
	Increased polycystic ovary tissue symptoms	*In vivo*, Wistar rats	100, 150, and 200 mg/kg – 60 days	Hydroalcoholic extract
	Preventing memory loss using the Morris Water Maze Test	*In vivo*, Alzheimer's Wistar rats	100, 150, and 200 mg/kg – 21 days	Hydroalcoholic extract
	Decreased levels of TG,HDL-C,VLDL-C,TG,AI, and CHD	*In vivo*- cholesterol-fed rabbits	0, 1, and 3% v/w of AW dissolved in 10 ml of distilled water – 28 days	Aromatic water
	Decreased levels of ALT and TG	*In vivo*, Wistar rats	400, 800, and 150 mg/kg - 21 days	Hydroalcoholic extract
	Accelerated wound healing, increased number of neutrophils and macrophages, and decreased number of fibroblasts over time	*In vivo*, Wistar rats with peptic ulcer	75 and 100 mg/kg - 14 days	Aqueous extract
	Reduced pain	Male rats	400, 800, and 1600 mg/kg	Hydroalcoholic extract
	Reduced immobility period in FST	Immobility, swimming and climbing behaviors in forced FST and TST using animal models of depression	50, 75, and100 mg/kg	Hydroalcoholic extract
	Protection against CPF-induced liver and lung tissue damage	Against chlorpyrifos (CPF)-induced liver and lung toxicity in rats	40, 80, and 160 mg/kg. 8 weeks	Hydroalcoholic extract
	Decreased activity of liver enzymes, oxidative stress, malondialdehyde, and increased levels of glutathione and superoxide dismutase	Cadmium-induced hepatotoxicity in male rats	100, 300, and 600 mg/kg	Hydroalcoholic extract
	Significant antibacterial effects of the extract on Staphylococcus aureus and S. epidermidis indicating inhibition zone diameters of 30 and 26 mm)	Antibacterial /activity	500, 250, 125, and 62.5 mg/ml	Ethanolic extract
	Antimicrobial effect against Escherichia coli, Bacillus subtilis, and S. aureus	Antibacterial activity	10 µl of extract per well	Ethanolic extract
	Less sensitivity of Pseudomonas aeruginosa and Enterococcusfaecalis to the oil and bactericidal effect of the oil on P. aeruginosa, E. faecalis, and Bacillus Cereus	Assessing antimicrobial activity by micro broth dilution assay	Essential oil	Essential oil
	Improvement of useful gut microflora (Lactobacillus) and a decrease in the harmful bacteria (coliform) in the ileum of broilers.	Adding oil to diets of broiler chicken	0.15% and 0.3% of diet	Mixed thyme and Ziziphora essential oil
	Stronger inhibitory and bactericidal effects of essential oil than that of the standard drug chlorhexidine	Candida albicans	Essential oil	Essential oil
	Inhibiting the growth of bacteria at low concentrations	Bacteria isolated from urogenital tract infections	10 µl/disk	Essential oil and methanolic extract
Bahareh et al., 2018	Strong antibacterial effect against E. faecalis	E. faecalis	Essential oil	Essential oil
	The effectiveness of Z. tenuior extract and its fractions against protoscolices and its anti-inflammatory properties	Protoscolices	10 mg/ml	Extract and its fractions (ethanol, petroleum ether, ethyl acetate, and chloroform)
	High scolicidal activity produced by ethanolic extract of Z. tenuior as an appropriate and effective scolicidal agent in hydatidosis surgeries	*In vitro *protoscolicidal effect	3-100 mg/ml for 10-60 min	Ethanolic extract
	Strong antimicrobial activity of alcoholic extract against many food pathogenic bacteria and its use as a natural antimicrobial in food products	Significant foodborne pathogens in vitro	10, 20, 30, and 40%	Methanolic extract
	Strong anti-toxoplasmic activity	Propagated tachyzoites of Toxoplasma gondii in the peritoneum cavity of mice	50,100, and 200 μg/ml	Ethanol extract
	Reduced number of amastigotes in macrophages	Cell lines (macrophage) were infected by promastigotes of Leishmania	5, 10, 15, 20, and 30 mg/ml	Aqueous and ethanolic extracts

**Effect on polycystic ovary syndrome**

This herb has anti-inflammatory effects due to the presence of pulegone, flavonoids, and anthocyanins. The anti-inflammatory properties of this extract were examined on the hormonal profile and the improvement of the tissue symptoms of polycystic ovary syndrome (PCOS) in rats. The findings showed significant decreases in the thickness of granulosa layer, the number of corpus luteum, and cyst formation, as well as an increase in C-reactive protein (CRP), in the PCOS group compared to the control group (that received no drug injection or extract treatment). However, tissue changes in the ovaries of the experimental groups (that received the extract or estradiol valerate) exhibited no significant difference compared to the control group. *Luteinizing hormone* (*LH*), estradiol, testosterone, and CRP levels decreased in the experimental groups. Regarding the anti-inflammatory effects, *Z. tenuior *extract could induce ovulation by improving the PCOS tissue symptoms and modulating hormone levels. In this study, the researchers also observed increased serum testosterone and estradiol levels and decreased serum progesterone levels caused by decreased corpus luteum formation and increased number of non-ovulated follicles and cysts. With respect to the inhibitory effects of flavonoids, thymol, and carvacrol on cyclooxygenase enzymes, this extract may play a role in improving ovarian tissue symptoms in PCOS rats treated with hydroalcoholic extracts by inhibiting the proliferation of theca layer cells (Table 1) (Nabiuni et al., 2015 ▶).

**Effect on the immune system**

CD40 is one of the co-stimulatory markers existing on dendritic cells, the surface expression of which is increased by *Z. tenuior *extract. However, it failed to change CD86 and MHC-11 molecules and consequently promote the development of dendritic cells (DCs). This extract increased the interleukin 12 (IL-12) production in DCs, inhibited the proliferation of allogenic T cells, and increased IL10 levels in allogeneic T cells. It can regulate the immune response by inducing the CD40 expression on dendritic cells and the production of cytokines. On the other hand, this extract inhibits the stimulatory activity of T cells at high concentrations; hence, it is probably effective in treating the immune system diseases (Azadmehr et al., 2014 ▶). The effects of different concentrations of the extract on the viability of rats’ peritoneal macrophages, their nitroxide and *Reactive Oxygen Species* (*ROS*) production, and antifungal activities were evaluated. Moreover, the aqueous extract of this herb enhanced the activities of macrophages. The extract also had a significant inductive effect on ROS levels and the antifungal activities of macrophages (Naeini Aet al., 2010 ▶).

**Antitumor effect**

The bioactive compounds of the *Z. tenuior *extract have antitumor activity and decrease the growth of malignant (32.6%) and cancerous (47.5%) tumors (Mohammadi and Mohammadi Mehdiabadi Hassani, 2017 ▶; Franks, 1995 ▶). 

**Effect on tyrosinase**

Tyrosinase plays a critical role in the melanin biosynthesis in skin pigments. The inhibitory Effect ofthis enzyme was evaluated through the effects of extracts from *Z. tenuior*, sage (*Salvia officinalis*), *Teacriumpolium*, and myrtle (*Myrtuscommunis*) on tyrosinase. All the extracts inhibited this enzyme in a dose-dependent manner. The maximum inhibitory effect was observed for the extract obtained from myrtle, which was comparable to hydroquinone as a standard drug. Afterwards, *Z. tenuior*, *Teacriumpolium*, and sage extracts had the highest inhibitory effects, respectively (Rafati, 2015 ▶). 

**Antioxidant properties**

The effect of a herbal tea containing *Z. tenuior *on the total antioxidant capacity (TAC), total thiol, and total MDA (oxidative stress indices) was evaluated in inactive girls after having a session of athletic activity, and the findings indicated that drinking a high amount of this herbal tea might increase TAC. The possible mechanism for the effect of this herbal tea on increasing TAC was explained by enhancing intracellular antioxidants such as bilirubin, uric acid, and albumin. In addition, some changes in MDA and no change in the total thiol suggested that the antioxidants could balance the antioxidant defense system (Rostami et al., 2017 ▶; Aliakbarlu and Shameli, 2013 ▶; Dakah et al., 2014 ▶; Mahdavi and Nobakht, 2018 ▶).

**Antimicrobial properties**

Aromatic herbs have long been used to prevent and treat infectious diseases, and they have recently attracted much attention. Researchers evaluated the antibacterial effect of ethanolic extracts from* Z. tenuior *and ajowan (*Trachyspermumcopticum *L*.*) on Gram-positive (four species) and Gram-negative (five species) bacteria. Both ethanolic extracts had significant antibacterial effects on the concerned bacteria. *S. aureus* and *S. epidermidi* were the most sensitive bacteria in response to these extracts, and Gram-negative bacteria were more resistant than Gram-positive ones (Kouhsari et al., 2014 ▶). The ethanolic extract from *Z. tenuior *had an inhibitory effect on the growth of Escherichia coli (E. coli), Bacillus subtilis, and *Staphylococcus aureus*. The chemical composition of *Z. tenuior* extract and of course its antimicrobial and pharmacological effects are affected by factors such as climatic characteristics, soil type, altitude and so on. (Najafi and Tavakkoli, 2011 ▶). Mahboubi et al. (2012) ▶ identified 44 compounds in the essential oil of this herb, the most significant of which were alpha terpineol, thymol, and acetyl geranyl. The antimicrobial effects of the essential oil from this herb were also confirmed on saprophytic fungi and *Pseudomonas aeruginosa*, Enterococcus faecalis, and *B. cereus *(Mahboubi et al., 2012 ▶).

Salman et al. (2017) ▶ studied the antifungal effects of essential oils obtained from native herbs, including *Prangosfrulacea*, *Z. tenuior*, *Ferula gummosa,* and *Dracocephalummoldavica*on *C. albicans* (yeast). Except for *F. gummosa*, the inhibitive and lethal effects of the essential oils were stronger than chlorhexidine (standard), and *Z. tenuior* had higher antifungal effects than the other herbal essential oils (Salman et al., 2017 ▶). In addition, *Z. tenuior* extract had high scolicidal activity and was used as an effective scolicidal agent in surgeries on hydrated cysts (Shahnazi et al., 2016a ▶; Shahnazi et al., 2017 ▶; Shahnazi et al., 2016b ▶). *Z. tenuior* was reported to be effective in controlling and treating leishmaniosis and affected the number of amastigotes appeared in macrophages (Hejazi et al., 2018 ▶).

**Chemical compounds in** ***Z. tenuior*** **extract and factors affecting the contents of these compounds**

Najafi and Tavakoli (2010) ▶ examined and compared the composition of the essential oil from *Z.tenuior* in two regions of Iran (Karaj and Qom) using gas chromatography/mass spectrometry (GC/MS). The results indicated that the quality of the essential oil and its antibacterial properties were affected by changes in the concentrations of elements existing in the soils of the two regions (Najafi and Tavakkoli, 2011 ▶). Moreover, climatic and ecological factors independently or simultaneously affect the morphological characteristics of this herb and raise diversity in its morphology. Among these factors, soil type had the lowest effect and the climate and altitude had the highest effects on the vegetative and reproductive organs of the herb (Talebi et al., 2010 ▶). In another study, the volatile components of the herb (*Z.tenuior*) were studied in two different regions of Turkey. The components identified in the herb from the two regions had 99.4-98.8% similarity, and their main component was pulegone (Sezik et al., 1991 ▶). Accordingly, the different effects of this extract on various body organs and its different antibacterial activities can be attributed to the type of soil and the altitude of the harvest region. Moreover, the high genetic diversity among this family of *Ziziphora *can be useful to apply heterosis in modifying and increasing bioactive compounds of this herb. Given the herb overharvesting, the establishment of its germplasm bank and propagation of this species were recommended in previous studies (Hatari et al., 2013 ▶; Navid et al., 2017).

The chemical analysis of the essential oil from *Z.tenuior *in Shahr-e Babak, Kerman, also showed that its main compounds were pulegone, 3',5'-dihydroxy acetophenone, isomentone, 2-metyl-5- (1-methy-ethyl) -phenol, limonene, 12 acetyl – 4, and 4 d-methyl-cyclopentine-2-isen (Pakniyat and Mousavi, 2014 ▶). Ziziphorins (A and B) are one of the new flavonoids extracted from *Z.tenuior *(Mehmood et al., 2010 ▶), and the aerial parts of this herb contain anthocyanins, proteins, and oils (Zarei et al., 2014 ▶).

Different extraction techniques of essential oil affect the quantity and quality of the oil. In a study, four different techniques,* viz*. simultaneous distillation extraction (SDE), hydro distillation (using a Clevenger-type apparatus), steam-cooled distillation (ultrasonic), and steam distillation, were used to extract the essential oil from two species (*Z.tenuior* and *Z. clinopodiodes)*. A high percentage of menthol in the essential oil from *Z. clinopodiodes* resulted in its high quality. The SDE was found to be the best method for the essential oil extraction in both herbs (Batooli et al., 2012 ▶).

## Discussion

Pulegone is one of the main compounds in *Z. tenuior, *which has analgesic and anti-inflammatory effects and reduces the gastric acid secretion. *Z. tenuior* extract affects serotonergic and opioidergic systems and consequently leads to analgesic activities. In this regard, its anti-inflammatory effects are comparable to dexamethasone. Flavonoids reduce pain by affecting GABA, opioid, and adrenergic receptors (Kouhsari et al., 2014 ▶; Zendehdel and Babapour, 2010 ▶). The extract contributes to wound healing by affecting the number of macrophages, neutrophils, and fibroblasts in the wound area. Over time, it increases neutrophils and macrophages and reduces the number of fibroblasts (Mohammadi and Mohammadi Mehdiabadi Hassani, 2018 ▶). This herb inhibits cholesterol absorption in the intestine, regulates LDL receptor, increases cholesterol and acidic bile excretion in the stool, and decreases cholesterol synthesis, thereby inducing a hyperlipidemic effect and weight loss. Thymol and carvacrol in this plant decrease the level of cholesterol in animals by inhibiting HMG-CoA reductase. Considering its antioxidant activity, this extract improves oxidative stress in the liver and lungs, enhances the activity of antioxidant enzymes in the body, and reduces MDA (Karimi et al., 2013 ▶). It has antidiabetic effects (Tomczyk et al., 2019 ▶) by increasing the number of pancreatic beta cells and insulin secretion and prevents memory loss in diabetic rats. *Z. tenuior* extract leads to skin whitening by inhibiting tyrosinase (Rafati, 2015 ▶). Although the extract has no side effects, a high amount of pulegone and its metabolites may raise pulmonary edema, internal bleeding, and hepatotoxic effects (Karimi et al., 2013 ▶; Anderson et al., 1996 ▶). 

## Conflicts of interest

The authors have declared that there is no conflict of interest.
